# The NFKB1 polymorphism (rs4648068) is associated with the cell proliferation and motility in gastric cancer

**DOI:** 10.1186/s12876-015-0243-0

**Published:** 2015-02-14

**Authors:** Ying Chen, Renquan Lu, Hui Zheng, Ran Xiao, Jingjing Feng, Hongling Wang, Xiang Gao, Lin Guo

**Affiliations:** 1 Department of Clinical Laboratory, Shanghai Cancer Center, Fudan University, Shanghai, 200032 China; 2 Department of Oncology, Shanghai Medical College, Fudan University, Shanghai, 200032 China

**Keywords:** NFKB1, Polymorphism, Gastric cancer, Susceptibility, Single nucleotide polymorphism

## Abstract

**Background:**

We have demonstrated previously that NFKB1 single nucleotide polymorphism (SNP) rs4648068 GG homozygote was associated with the increased risk of gastric cancer in Chinese Han population. In this study, we constructed the recombinant plasmid pGL3-AA, pGL3-GG, pGL3-AA-NFKB and pGL3-GG-NFKB to investigate the function of rs4648068 by cell biology experiments.

**Methods:**

Quantitative real-time PCR was used to detect NFKB1 SNP rs4648068 genotype in the patients with gastric cancer. Anti-NF-κB1 p50 polyclonal antibodies were used for immunohistochemical analysis of the tissue specimens. The subsection of NFKB1 containing the promoter site and adjacent three consecutive exons were obtained by PCR technique and subcloned into the vector pGL3-Basic. Dual-Luciferase reporter assay was used to detect the transcriptional activity of the constructed promoter. Effects of transcription factor NFKB1 on C/EBPβ expression were determined by chromatin immunoprecipitation and Western analysis. Furthermore, proliferation and invasion ability of the transduced cell were also measured and compared.

**Results:**

Intensive staining for p50 expression was observed in the tissues of GG genotype patients, compared with those of GA group and AA genotype patients. The transcriptional activity of rs4648068 (A > G) by dual-Luciferase reporter assay suggested that the luciferase activity of homozygote group (pGL3-GG) was greater than that of the control (pGL3-AA), especially at the stimulation of LPS. We found that the luciferase activity was also influenced by pGL3-GG levels. The effects of NFKB1 rs4648068 were enhanced by rs4648065 on the transduced cells. The interaction between NFKB1 promoter nucleotide sequence and C/EBPβ was regulated by the functional SNP rs4648068 in SGC-7901 cells. Our data indicated that the transduction of pGL3 expression plasmid pGL3-GG-NFKB improved the proliferation and motility of gastric cancer cells. Correspondingly, the homozygote GG of SNP rs4648068 strengthened the transcriptional activity of NFKB1 and influenced the cell biological activity.

**Conclusion:**

The transcriptional activity of NFKB1 was associated with SNP rs4648068, and this functional SNP site has the important effects on cell proliferation and motility.

## Background

Nuclear factor-κB (NF-κB) regulates many cellular functions including cell proliferation, apoptosis, angiogenesis, immune response, cell adhesion and differentiation [1]. The signal pathway involved by NF-κB plays important roles in linking inflammation to tumor development and progression [2]. NF-κB family contains five members in mammals: NFKB1 encoding p50, NFKB2 encoding p52, RELA encoding p65, REL encoding c-Rel, and RELB encoding Rel-B. The most common dimer is the p65/p50 heterodimer, which is inhibited by IκB in the cytosol [3]. Internal gene mutations and external stimuli contribute to the release of activated NF-κB [4]. The elevated NF-κB levels have been reported to be associated with cancers [5-7].

Rs4648068 polymorphism locates at NFKB1 promoter region. Polymorphisms in the regulatory regions of the genes have been associated with variation in gene expression [8,9]. The rs4648068 polymorphism is hypothesized to have a significant effect on NF-κB1 expression and play a part in tumorigenicity.

In the previous case–control study, we have demonstrated the role of NFKB1 rs4648068 polymorphisms in gastric cancer susceptibility in Chinese Han population. The results further indicated that the subjects with the allelic homozygous GG in rs4648068 had an increased risk in gastric cancer group compared with that in controls (*P* = 0.005) [10]. However, the motility-promoting function of this single nucleotide polymorphism (SNP) site in gastric cancer progression is still uncertain. To clarify the functional mechanisms of NFKB1 rs4648068 polymorphism, it is necessary to investigate the changes of NF-κB1 expression at cellular and molecular levels. Accordingly, the polymorphism rs4648068 might play a role in the development of gastric cancer.

In this study, we constructed the recombinant plasmids, containing the different SNP variants to investigate the changes on transcriptional activity of NFKB1. Additionally, the growth and invasion alteration of transduced cells was observed to further demonstrate the biological function of rs4648068 in gastric cancer.

## Methods

### Patients’ samples and cell lines

In previous study, we conducted a large-scale case–control study on Chinese Han population to investigate the association of rs4648068 polymorphism with gastric cancer or clinic-pathologic variables of gastric cancer patients. To further verify its function, the expression levels were measured with 20 tissues from gastric cancer patients using immunohistochemical staining. Meanwhile, the genomic DNA of these patients was collected to do genotyping. The study design was approved by the Clinical Research Ethics Committee of the Fudan University Shanghai Cancer Center. All of the participants signed informed consent documents prior to participating in this study. Paraffin-embedded gastric cancer tissue samples were collected from 20 patients with gastric cancer who were admitted to Fudan University Cancer Hospital (Shanghai, China) July 2014. All cases were gastric adenocarcinoma confirmed by pathologist. Only the patients whose clinical data (include diagnosis, age, sex, address, disease history, etc.) were intact were selected. The gastric carcinoma cell lines, SGC-7901 cells, MKN45 cells, HGC27 cells and the non-gastric cancer cell lines, 293 T, Hela cells, were generous gifts from Laboratory of Oncology, Fudan University Shanghai Cancer Center, Shanghai.

### Sample preparation and NFKB1 genotyping

Genomic DNA was extracted from peripheral blood samples of each study subject using the TIANamp Blood DNA Kit (Tiangen Biotech, China). NFKB1 SNPs rs4648068 were genotyped by SYBR allelic discrimination. The corresponding qRT-PCR specific primers pGL3-RTp1, pGL3-RTp2 and pGL3-RTp3 are listed in Table 1. PCRs were run in a 10 μL reaction solution containing 30 ng of template DNA, 5 μL SYBR Premix Ex Taq (TAKARA, Japan) according to the manual. PCR was performed at 95°C for 10 min and 40 cycles at 95°C for 30 s and 58°C for 30 s. The samples were amplified, read and analyzed in Option® Fluorescence Temperature Cycler (MJ Research, Canada).

**Table 1:** **Primers used in this study**

Name	Sequence	Restriction enzyme
pGL3-RTp1	5′ TAA TTG TTA GAG ATT CCA A 3′	
pGL3-RTp2	5′ TAA TTG TTA GAG ATT CCA G 3′	
pGL3-RTp3	5′ ACA ATG TTA GAT TTT ACC ATG ATT 3′	
pGL-promoterFor1	5′GG*GGTACC*GGTCATCCTAGATCGTACTAAG3′	KpnI
pGL-promoterFor2	5′GG*GGTACC*ATAAAAGAAGAGAGTGCTGGAG3′	KpnI
pGL-promoterRev	5′GA*AGATCT*CCTTGACATATCATTTTAGTTG3′	BglII
pGL-NFKBFor	5′GA*AGATCT*ATGACGCCCTTGCACTTGGCAGTGA3′	BglII
pGL-NFKBRev	5′GA*AAGCTT*TTAGCTGCTTTGAGAAGAGCT3′	HindIII

The restriction sites for primers are italics.

### Immunohistochemical staining

Immunohistochemical staining was performed on 6-μm sections from formalin-fixed, paraffin-embedded human tissues. The slides were deparaffinized and rehydrated ingraded ethanol solutions. Antigen retrieval was performed by heating the samples for 20 min in citrate buffer (pH 6.0). The slides were then incubated with a rabbit anti-NF-κB p50 polyclonal antibody (1:100 dilution; ab7971, Abcam) overnight at 4°C. Tissue sections were washed again in PBS and incubated with horseradish peroxidase-conjugated secondary antibodies for 60 min at 37°C. Color was developed using diaminobenzidine as a chromogen and the slides were counterstained with hematoxylin.

### Evaluation of immunohistochemical staining results

The NF-κB1 positive tissues were quantified based on the percentage of positive cells which were serially counted in one microscopic field. The cell counting was repeated in five random microscopic fields at × 400 magnification. Two pathologists who were blinded to patient group independently interpreted the IHC staining results using positive index (PI). The positive index (PI) was calculated using the following formulation: *PI = i × p*, where *i* is intensity of staining (0 for negative, blue; 1 for weakly-positive, light yellow; 2 for medium positive, yellow; 3 for strong positive, brown), and *p* is positive percentage of staining (1 for ≦10%; 2 for 11%-50%; 3 for 51%-75%; 4 for >75%) [11]. Then, the positive index (PI) was calculated for each case. If there were divergences in the PI determined by the two pathologists, slides were rescored until a consensus was reached. Besides, differences in NF-kappaB1 expression between different groups were investigated using Kruskal-Wallis non-parametric test.

### The construction of recombinant plasmid

The section containing NFKB1 promoter region with polymorphisms rs4648068 was obtained by PCR using primers pGL-promoterFor1 and pGL-promoterRev (Table 1). DNA templates were extracted from peripheral blood samples of gastric cancer patients using the method described above. The PCR products containing NFKB1 polymorphisms were digested by Bgl II and Kpn I and linked into the vector pGL3-basic (Promega, Madison, WI) to construct recombinant plasmid pGL3-AA and pGL3-GG. Meanwhile, pGL3-GG/TT containing rs4648065 site was established as above described method to investigate the co-effect on rs4648068. Furthermore, the sequence coding selected section of NF-κB1 was amplified to construct the NF-kappaB expression plasmid according to the human NF-κB neclotides sequences (NM_001165412.1) in GenBank. The CDS region containing adjacent three consecutive exons was amplified using pGL-NFKBFor and pGL-NFKBRev (Table 1), subcloned into pGL3-AA and pGL3-GG, named as pGL3-AA-NFKB and pGL3-GG-NFKB respectively, while the random DNA fragments and above PCR products was subcloned into the vector pGL3-basic to construct recombinant plasmid pGL3-mock. The recombinant plasmids pGL3-AA and pGL3-GG were constructed for luciferase assay, while the expression plasmids pGL3-AA-NFKB and pGL3-GG-NFKB were constructed for cell biological experiment. Non-gastric cell lines such as 293 T cells and Hela cells, gastric cancer cell lines such as MKN45 cells, HGC27 cells and SGC7901, were transfected with pGL3-AA and pGL3-GG using Lipofectamine 2000 (Invitrogen) respectively to verify the transcriptional activity of NFKB1 promoter. The expression plasmids were also transfected into SGC7901 cells, designated as 7901-pGL3-mock, 7901-pGL3-AA, and 7901-pGL3-GG. The PCR reactions were carried out at 94°C for 2 min, then a 3-step cycle procedure was used (denaturation at 94°C for 30s, annealing at 57.3°C for 40 s, and elongation at 70°C for 3.5 min) for 39 cycles, with a final extension at 72°C for 10 min.

### Transfection and luciferase assay

The luciferase assay is one of the most convenient reporter assays to explore the regulation of gene expression in mammalian cell culture. The pGL3-basic vector only has the luciferase gene and lacks regulatory regions, such as promoter sequences. This vector is useful in the study of functional promoter elements to regulate gene expression. Renilla luciferase pRL-SV40 vector was used to normalize and reduce differences in transfection efficiencies and subsequent variations in these experiments.

For our luciferase assays, approximately 7 × 10^4^ of SGC-7901 cells were cultured in a 24-well plate in antibiotic-free media. After attachment, cells were co-transfected with recombinant plasmids and pRL-SV40 vector by using Lipofectamine 2000 (Invitrogen, Carlsbad, CA). Other cell lines, such as 293 T cells, Hela cells, MKN45 cells and HGC27 cells, were transfected with these plasmids by the same method. LPS (100 ng/mL) was added into wells of stimulation group 24 h later. After 48 h incubation with cell culture media, then cells were lysed in passive lysis buffer (Promega, Wisconsin, USA). Firefly and Renilla luciferase signals were measured by the Dual-Luciferase® Reporter Assay System (Promega, Wisconsin, USA) in Synergy H4 Hybrid Microplate Reader (BioTek, Winooski, USA). Relative luciferase activity (Luc) calculated by the ratio of Firefly and Renilla luciferase signals was used to monitor the efficiency of transfection.

### Chromatin immunoprecipitation

Five million SGC-7901 cells were cross-linked with 1% formaldehyde at room temperature for 10 min. Chromatin extracts were obtained by cell lysis buffer. Sonication was performed 14 times for 4.5 s each at Power 25% (Thermo™, Ultrasonic Cell Disruptor, Model XL) resulting in DNA fragments between 150 and 1000 bp in size. Supernatants were collected and submitted to immunoprecipitation with 5 μg of rabbit anti-C/EBPβ polyclonal antibody (sc-150X, Santa Cruz Biotechnology) and 20 μl of magnetic protein A/G beads (Magna ChIP™ A/G, Millipore, USA) overnight at 4°C. In parallel, supernatants were incubated with normal rabbit IgG antibody as negative controls. Protein A/G magnetic beads were washed and pelleted by magnetic separator, and heated in ChIP Elution Buffer with Proteinase K at 65°C for 2 hours to reverse the cross-links of protein/DNA complexes. DNA fragments were purified on offered Spin columns. Finally, 4 μl from a 30 μl extraction were PCR amplified for NFKB1 promoter genes using the primers pGL-promoterFor1 and pGL-promoterRev (Table 1).

### Western blot analysis

The rabbit anti-p50, anti-GAPDH antibody and the secondary antibody were purchased from Abcam (Cambridge, MA, USA). The **C/EBPβ polyclonal antibody** was purchased from Santa Cruz Biotechnology (Santa Cruz Biotechnology, CA, USA). Cultured cells were washed with phosphate-buffered saline and lysed on ice for 30 min with 500 μL lysis buffer (Beyotime, China). The cell lysate was centrifuged at 4°C at 10,000 × g for 15 min. The supernatant nuclear protein was used for Western blot. Twenty micrograms of cell extracts was loaded into 12% sodium dodecyl sulfate-polyacrylamide gel electrophoresis and subjected to Western blot. GAPDH was used as the internal control. Enhanced chemiluminescence detection was performed according to the manufacturer’s instructions using X-photo film (Alpha Innotech, San Leandro, CA) and chemiluminescent substrate (Thermo Scientific, Rockford, IL).

### Cell proliferation and invasion assay

For the cell proliferation assay, 7901-pGL3-mock, 7901-pGL3-AA and 7901-pGL3-GG cells were grown at a density of 7 × 10^4^ cells/mL in 24-well plates with regular medium changes. The cell number was counted every 24 hours for 6 days as follows: cells were detached by brief exposure to 0.025% trypsin containing 2 mM EDTA in PBS, washed in culture medium without FBS, and then resuspended in the same medium for manual cell counting. For the invasion assays, matrigel was thawed at 4°C overnight, diluted in cold serum-free culture medium, plated onto 24-well plates preloaded with Transwell™ culture inserts (12 mm diameter, 8 μm pore size; Costar, Cambridge, MA, USA) and incubated for 5 h at 37°C for gelling. The cells were then plated onto the Transwell™ inserts (30,000 cells/well) and cultured at 37°C in 5%CO_2_. After 16 h, the cells on the upper side of the well were removed and fixed prior to staining with hematoxylin and eosin. Cells migrating to the underside were counted under a microscope. All experiments were repeated at least three times.

### Apoptosis assay

SGC-7901 cells were transfected with pGL3-AA-NFKB and pGL3-GG-NFKB respectively. At 48 h after transfection, cells were collected and resuspended in binding buffer containing Annexin V-PE and PI (BD Pharmingen, La Jolla, CA). Cell-associated fluorescence was analyzed with a FACS can instrument and associated Winlist 5 software.

### Statistical analysis

For immunohistochemistry *PI* of NF-κB1 expression, data were analyzed by Kruskal-Wallis non-parametric tests. The difference in the levels of luciferase reporter gene expression between different groups was determined by Student’s *t* test and One-way ANOVA analysis (SPSS version 13.0; SPSS Inc., Chicago). Differences between variants were considered significant at *P* < 0.05.

## Results

### Expression of NF-κB1 in gastric cancer tissues

**Figure 1:**
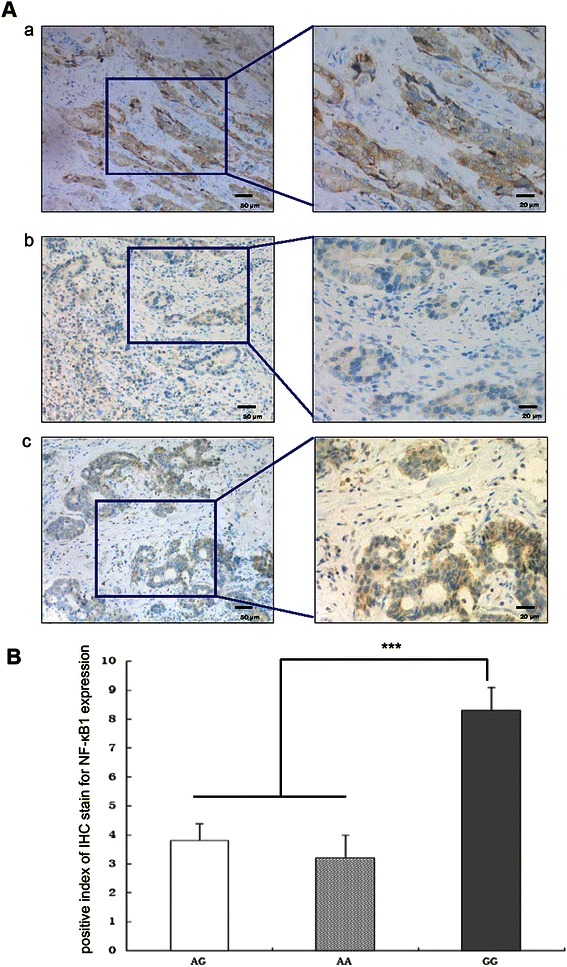
**Expression of NF-κB1 in gastric cancer tissues. A**. The staining results were shown. NF-κB1 (brown) is mainly expressed in cytoplasm of gastric adenocarcinoma tissue. (a) The patients’ genotype was GG. Dark brown NF-κB1 cytoplasm staining was observed. (b) The patients’ genotype was AA. NF-κB1 immunohistochemical staining of AA group exhibited faint. (c) The patients’ genotype was GA. NF-κB1 immunohistochemical staining was also faint. Left panels magnification, ×200; Right panels magnification, ×400. **B**. Differences in NF-kappaB1 expression between different groups were invcestigated using Kruskal-Wallis non-parametric test. The immunohistochemistry *PI* of NF-κB1 expression for GG group was significantly higher than those of GA group and AA group (*P* < 0.01). ***P < 0.01, **P < 0.05.

In previous study, we conducted a large-scale case–control study on Chinese Han population to investigate the association of rs4648068 polymorphism with gastric cancer or clinic-pathologic variables of gastric cancer patients. To further verify its expression profile, 20 tissues from gastric cancer patients were analyzed by immunohistochemical staining. The corresponding genomic DNA of these patients was also performed genotyping analysis. Our data showed that the NF-κB1 protein p50 was located in both the cytoplasm and nuclei of gastric cancer tissues. Diffuse staining for p50 was observed in the tissues of GG genotype patients, compared with the GA group and the AA group (Figure 1). The immunohistochemistry *PI* of NF-κB1 expression for GG group was significantly higher than those of GA group and AA group, analyzed by Kruskal-Wallis non-parametric test (*P* < 0.01).

### The construction and identification of recombinant plasmid

**Figure 2:**
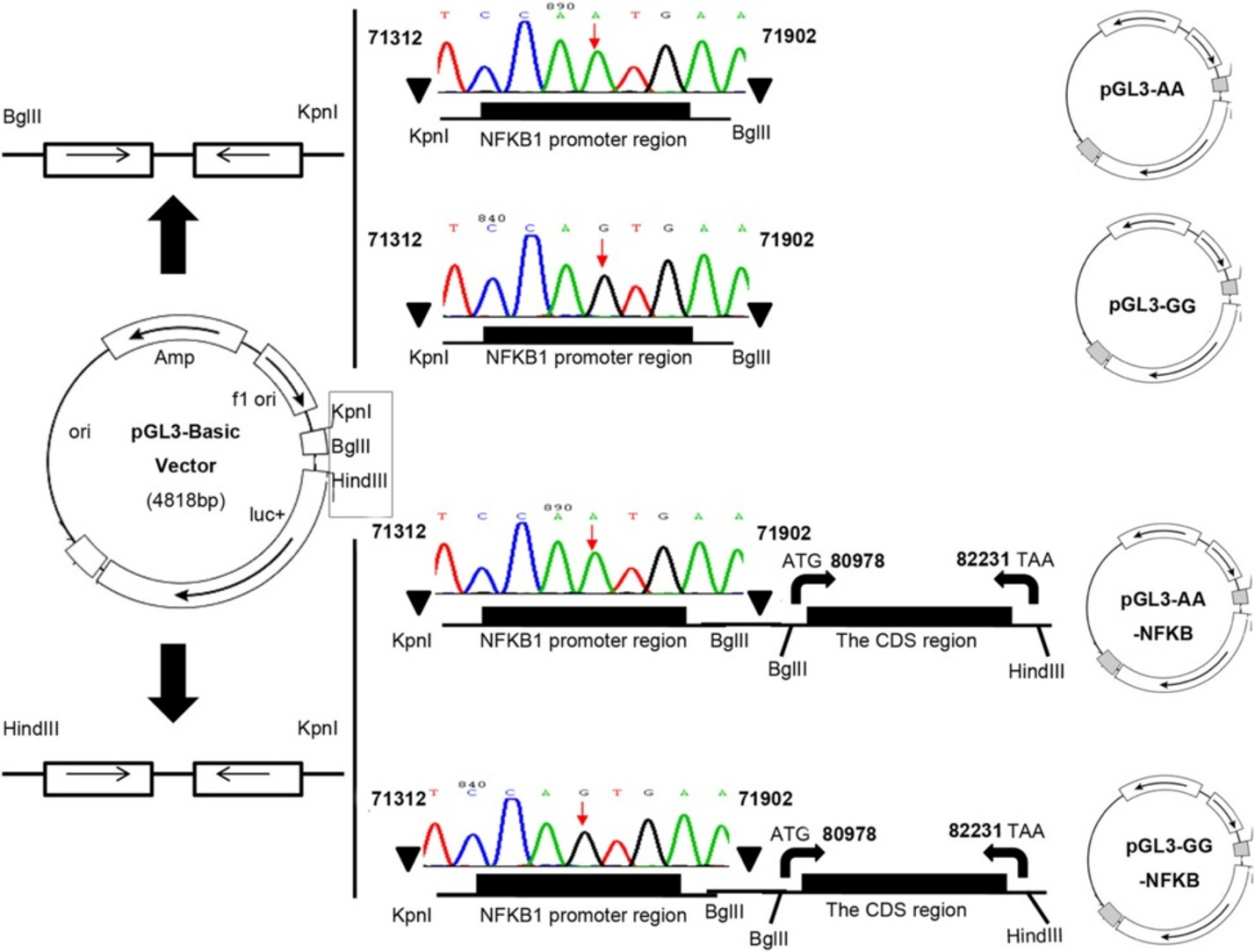
**The recombinant plasmid was identified by nucleotide sequence.** Map showed that the promoter region and three consecutive exons for NF-κB1 were subcloned to the pGL3-basic vector. Triangles above the map indicate the position of NF-κB1 promoter, and the bent arrows indicate the three exons for NF-κB1 (from ATG to TAA, altogether 1254 bp). The sequence of recombinant plasmid was correct without base mutation and deletion, and the sequence inserted was corrected by comparing with GenBank.

The promoter region containing NFKB1 polymorphisms rs4648068 (A > G) was subcloned into the vector pGL3-basic successfully. The recombinant plasmids were named as pGL3-AA and pGL3-GG. In addition, pGL3-GG/TT containing SNP rs4648065 (C > T) was established using the same method to investigate the co-effect on rs4648068. Furthermore, adjacent three consecutive exons for NF-κB1 was amplified and subcloned into pGL3-AA and pGL3-GG respectively, named as pGL3-AA-NFKB and pGL3-GG-NFKB. Nucleotide sequence further confirmed that the sequence of recombinant plasmid was correct without base mutation and deletion, and the sequence inserted was corrected by comparing with GenBank as is shown in Figure 2. Subsequently, we confirmed that the recombinant luciferase could be expressed in the transduced cells.

### Gene expression activity was associated with NFKB1 polymorphisms

**Figure 3:**
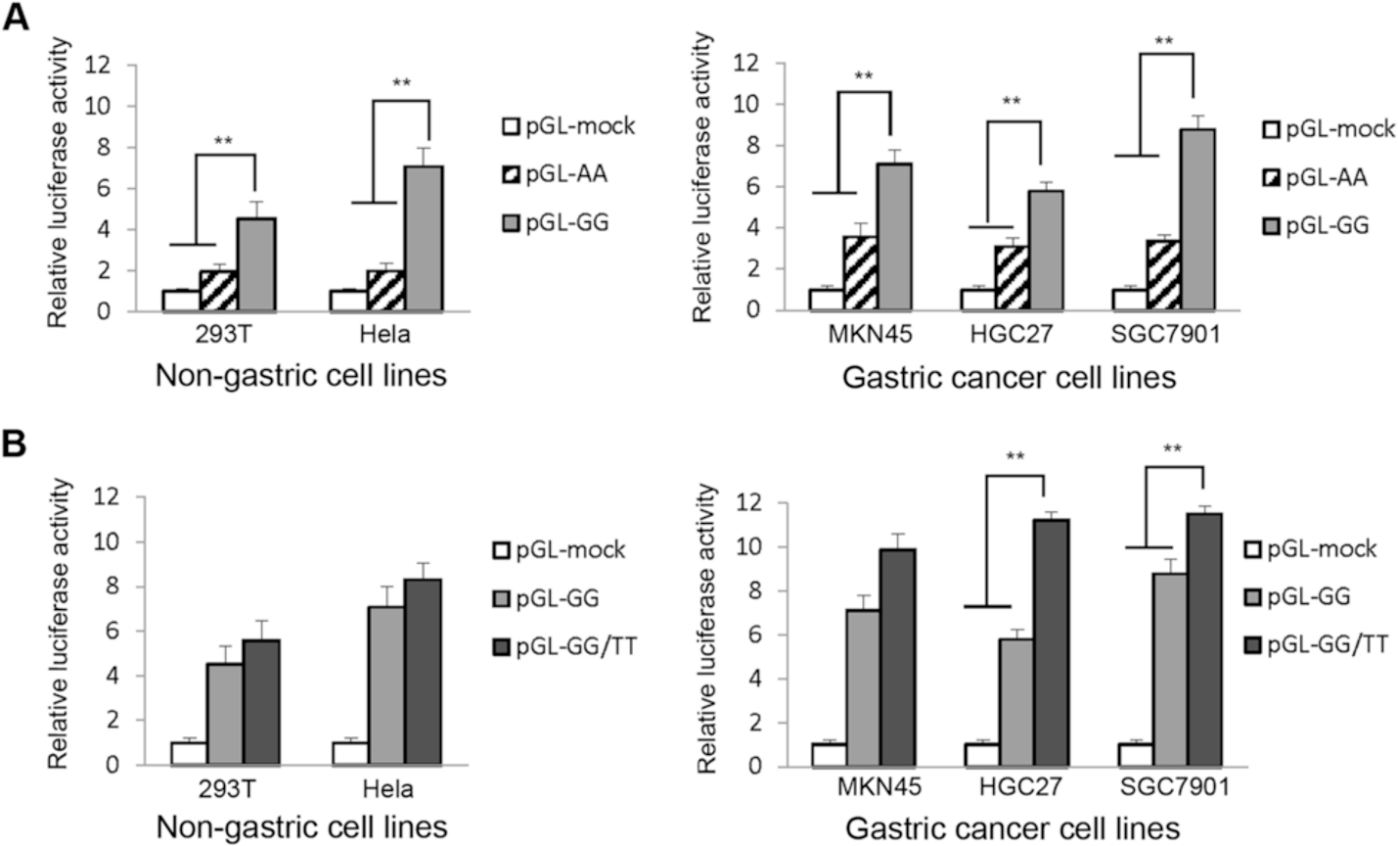
**NFKB1 polymorphisms significantly influenced luciferase gene expression in various cell lines. A**. Luciferase activity in various cell lines transfected with recombinant plasmid (pGL3-GG) was compared with control group (pGL3-mock and pGL3-AA). **B**. Luciferase activity in cell lines transfected with (pGL3-GG/TT) was assessed to identify the synergistic effect by rs4648065. Data are representative of at least three independent experiments. The difference was determined by One-way ANOVA analysis (SPSS version 13.0; SPSS Inc., Chicago). **P < 0.05.

To examine the functional activity of NFKB1 promoter variations A > G (rs4648068), as well as the synergistic effect by C > T (rs4648065), which was described in our previous study, different recombinant plasmids containing homozygote site were transiently transduced for transcriptional activity evaluation, 48-hours after transfection, renilla and firefly luciferase activities were measured. For non-gastric cell lines, luciferase activity for GG homozygote (4.53 ± 0.81) were higher than that of AA homozygote (1.94 ± 0.37) in 293 T cells (*P* = 0.041). In Hela cells, the luciferase activity for GG homozygote (7.09 ± 0.91) were also greater, compared with that of AA homozygote (1.97 ± 0.38, *P* = 0.002). For gastric cell lines, the luciferase expression ratios (7.12 ± 0.68 for MKN45 cells, 5.80 ± 0.42 for HGC27 cells, and 8.80 ± 0.64 for 7901 cells) in GG homozygote were observed higher than AA homozygote (3.56 ± 0.67 for MKN45 cells, 3.09 ± 0.43 for HGC27 cells, and 3.36 ± 0.30 for 7901 cells; *P* = 0.022, *P* = 0.04 and *P* = 0.004, respectively) with significant difference (Figure 3A). Moreover, two homozygote GG/TT panel for gastric cell lines (11.23 ± 0.38 for HGC27 cells, and 11.52 ± 0.15 for 7901 cells) showed stronger luciferase activity than that of homozygote GG panel (5.80 ± 0.42 for HGC27 cells, and 8.80 ± 0.64 for 7901 cells; *P* = 0.001, *P* =0.038 respectively) (Figure 3B). The result showed that the luciferase activity of homozygote group (pGL3-GG) was greater than control group (pGL3-AA) (*P* <0.05) (Figure 3A). Moreover, the luciferase activity of two homozygote site group (pGL3-GG/TT) was the strongest with significant difference in Figure 3B (*P* <0.05). The difference was determined by One-way ANOVA analysis (SPSS version 13.0; SPSS Inc., Chicago). It indicated that SNP rs4648068 significantly influenced luciferase gene expression in various cell lines. In addition, we found that the effect of NFKB1 polymorphism rs4648068 was enhanced by polymorphism rs4648065.

### The effect of NFKB1 rs4648068 influenced by LPS stimulation

The bacterial product LPS plays a role in the NF-κB signaling pathway. Thus, we investigated the effect of NFKB1 rs4648068 on LPS-stimulated luciferase activity. Both of recombinant plasmid pGL3-AA and pGL3-GG containing the promoter region were transduced to SGC7901 cells. The difference between luciferase activities of GG and AA reporters was estimated under the stimulation of LPS (Table 2). In the control group without LPS stimulation, the relative luciferase activity of pGL3-GG was greater than that of pGL3-AA, and the most effective concentration of recombinant plasmid was 100 ng/mL. Under the stimulation of LPS, the increased range of relative luciferase activity was elevated greatly. To compare the luciferase activity, we found that the increased range in LPS-stimulated group was more obvious than the control group with significant difference, analyzed by Student’s *t* test (Table 2). It suggested that the SNP rs4648068 can enhance the transcriptional activity of NFKB1, especially in LPS response.

**Table 2:** **The increased luciferase activity in LPS-stimulated SGC-7901 cells and control cells**

	The increased luciferase activity^Δ^of recombinant plasmid			
**Group**	25 ng/mL	50 ng/mL	100 ng/mL	150 ng/mL
**Control**	0.34 ± 0.03	0.91 ± 0.23	1.77 ± 0.53^**^	1.19 ± 0.36
**LPS**	2.86 ± 0.17^#^	4.54 ± 0.19^#^	5.32 ± 0.27^#^	5.74 ± 0.44^#^

Control group, unstimulated transduced cells; ^Δ^, increased Luc = Luc (GG)-Luc (AA); ^#^, *P* < 0.01, compared with control group respectively (Student’s *t* test); **, *P* < 0.05, compared with other concentration levels (25 ng/mL, 50 ng/mL and 150 ng/mL) (One-way ANOVA.).

### Effects of polymorphisms on protein expression

**Figure 4:**
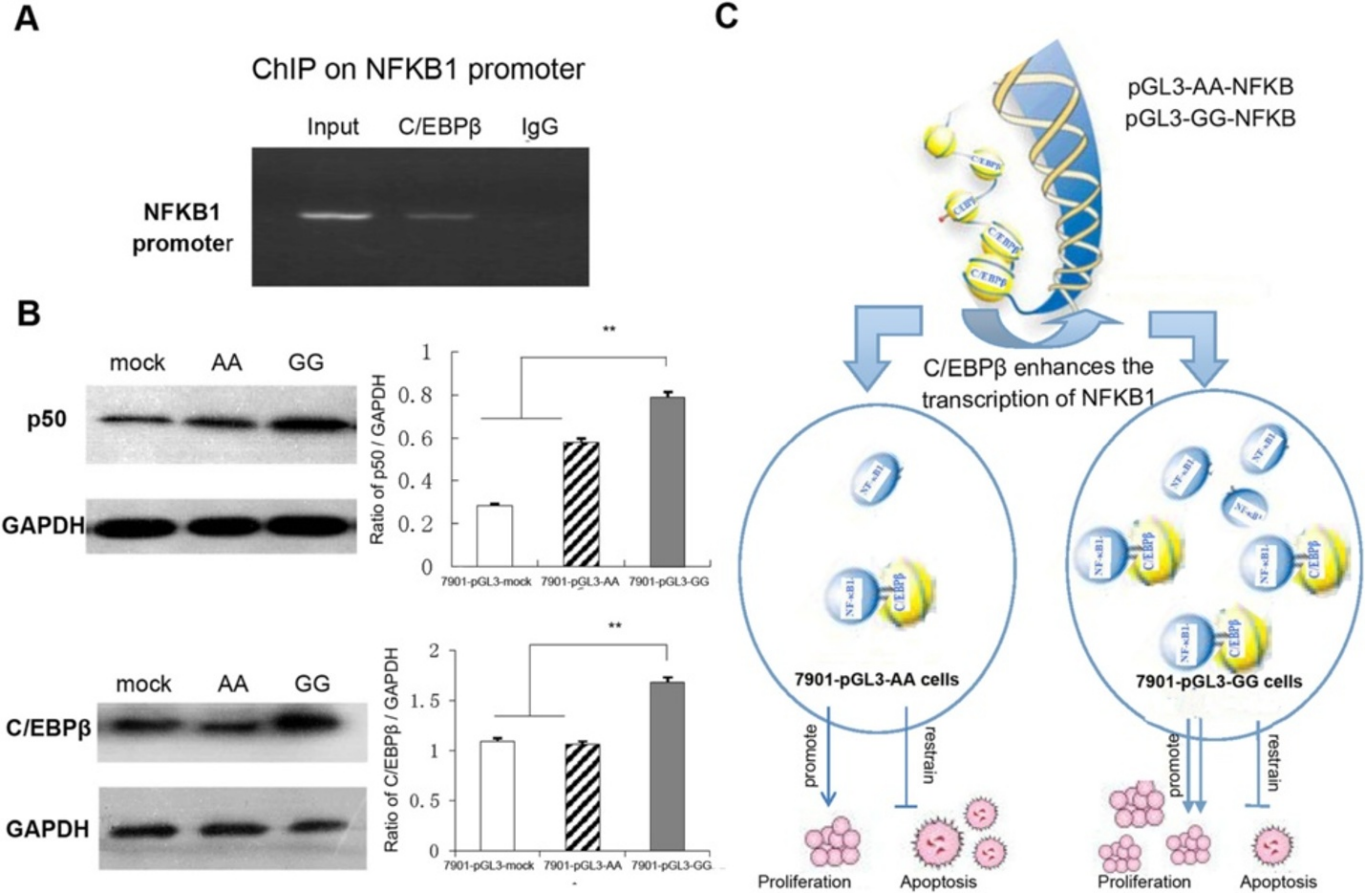
**NFKB1 polymorphisms influenced protein expression in plasmid transfected SGC-7901 cell. A**. A representative gel result of ChIP of C/EBPβ on the NFKB1 promoter was shown. **B**. Western blotting showed a higher level of NF-κB1 and C/EBPβ protein expression in 7901-pGL3-GG cells, compared with 7901- pGL3-AA cells. The gray value was analyzed by One-way ANOVA. **P < 0.05. **C**. A diagram was shown to clarify the interaction between NFKB1 promoter and C/EBPβ.

C/EBPβ (CAAT/enhancer binding proteins) is a transcription factor that can bind as a homodimer to certain DNA regulatory regions. To further define the role of rs4648068, chromatin immunoprecipitation assay (ChIP) was used to investigate the interaction between C/EBPβ and NFKB1 promoter in the SGC-7901 cell. The recombinant plasmid pGL3-AA-NFKB and pGL3-GG-NFKB, which contain the promoter region of NFKB1 and adjacent three consecutive exons, were transduced to the SGC-7901 cells, recognized as 7901-pGL3-AA cells and 7901-pGL3-GG cells. We found, as expected, that C/EBPβ bound to the NFKB1 promoter containing the SNP site (Figure 4A). Meanwhile, western blotting showed a higher level of p50 and C/EBPβ expression in 7901-pGL3-GG cells, compared with those of 7901-pGL3-mock cells and 7901- pGL3-AA cells (Figure 4B). It seems that C/EBPβ binds better to the GG version of NFKB1 promoter and enhances the transcription of NFKB1. On the other hand, NF-κB1 p50 could bind C/EBPβ and reciprocally induce partner’s expression, forming a transcriptional positive feedback loop, contributing to the upregulated p50 and C/EBPβ expression (Figure 4C). Since NF-κB1, as well as C/EBPβ, plays a role in the cell proliferation, differentiation, signaling pathway and the occurrence of tumors. The SNP site rs4648068 might have effect on cell proliferation and motility by regulating NF-κB1 and C/EBPβ. However, the mechanism for regulating C/EBPβ expression remains unclear, and will be performed in our future work.

### Proliferation and invasion ability of transfected SGC-7901 cells

**Figure 5:**
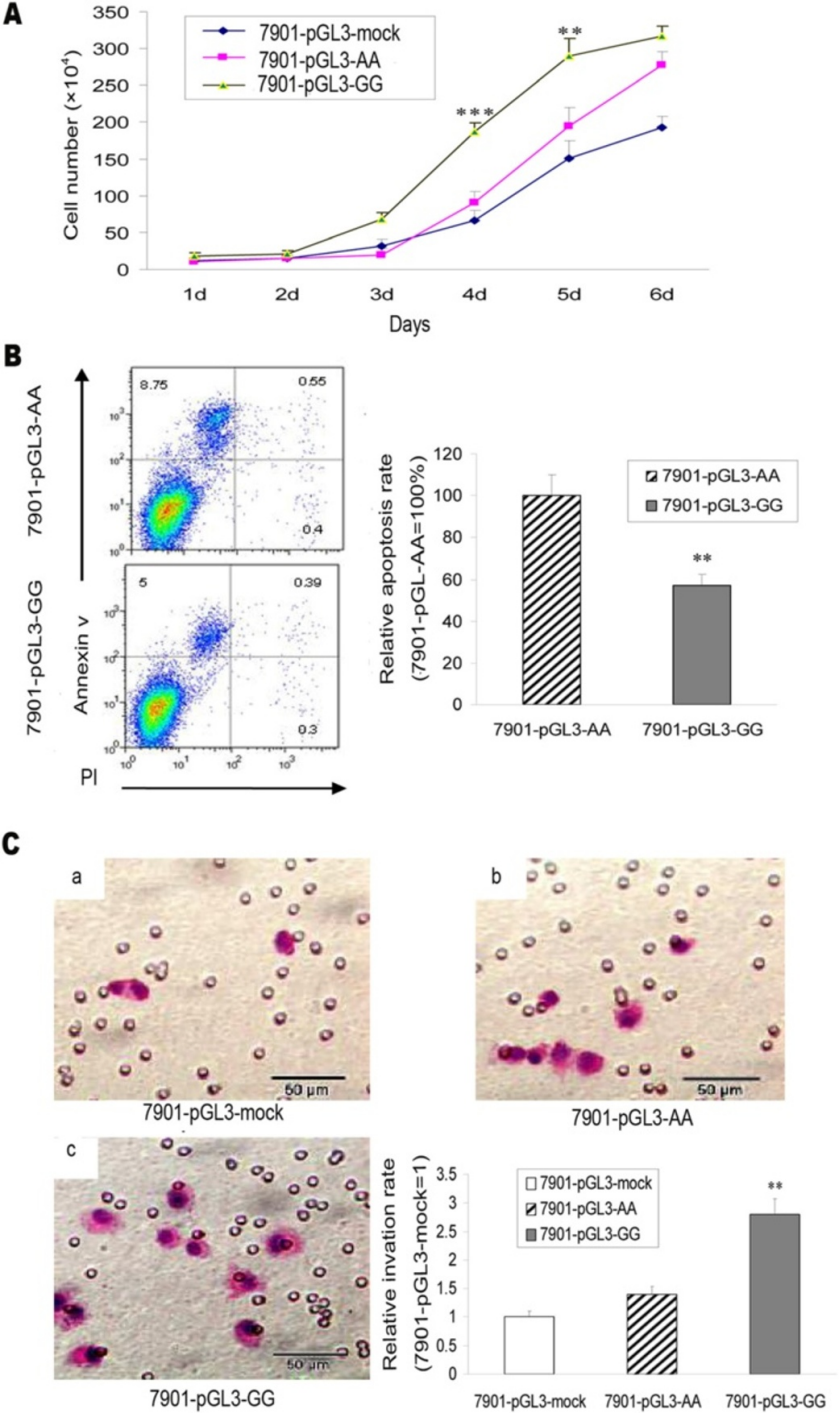
**The SNP rs4648068 influenced cell proliferation, apoptosis percentage and invasive ability. A**. Cell proliferation was significantly elevated in 7901-pGL3-GG cells compared with 7901-pGL3-AA cells and 7901-pGL3-mock cells. **B**. In each box, the lower left corner included viable cells, which were both Annexin V-FITC and PI negative. In the upper left corner, cells are in early apoptosis with Annexin V-FITC positive and PI negative. While the upper right corner included necrotic or late apoptotic cells are both FITC Annexin V and PI positive. In 7901-pGL3-GG cells, the percentage of apoptosis cells decreased significantly, compared with 7901-pGL3-AA. **C**. The invasion ability was increased in 7901-pGL3-GG cells (c) than control cells (a, b). 200×. a, 7901-pGL3-AA cells; b, 7901-pGL3-mock cells; c, 7901-pGL3-GG cells. Data were assessed by One-way ANOVA analysis. ***P < 0.01; **P < 0.05.

To further analyze the cell biological function of SNP rs4648068, we conducted proliferation and invasion assay in transfected SGC-7901 cells. Proliferation was assessed by cell counting under an optical microscope for six days. The proliferative rate of 7901-pGL3-GG cells was observed faster than 7901-pGL3-AA cells and 7901-pGL3-mock cells (Figure 5A). For the invasion assay, 7901-pGL3-GG cells showed a higher ability to migrate, compared with 7901-pGL3-AA cells and 7901-pGL3-mock cells (Figure 5C). It illustrated that the proliferation and invasiveness of SGC-7901 was enhanced by transfection of the recombinant plasmid containing SNP site. To guarantee the effective transduction, all the experiments were completed within six days after the transfection.

### Apoptosis assay

SGC-7901 cells were transfected with pGL3-AA-NFKB and pGL3-GG-NFKB respectively. At 48 h after transfection, cells were collected and resuspended in binding buffer containing Annexin V-FITC and PI, and then processed for flow cytometry analysis. In 7901-pGL3-GG cells, the percentage of apoptosis cells decreased significantly, compared with 7901-pGL3-AA cells (Figure 5B). The result demonstrated that in certain situations NF-κB1 acts as an anti-apoptotic protein.

## Discussion

Our previous study indicated that NFKB1 polymorphism at intron region (rs4648068) was associated with increased risk of gastric cancer in Chinese Han population. The present study focused on the investigation of NFKB1 transcriptional activity and biological function influenced by rs4648068 (A > G) polymorphism.

It was reported that NF-κB is constitutively activated in gastric carcinoma tissue [12]. Kwon HC et al. [13] demonstrated that the expression of NF-κB is increased in human gastric cancer tissue. The nuclear translocation of Rel A (used as a marker of NF-κB activation) was significantly greater in gastric cancer cells than in adjacent normal epithelial cells [14]. Yamanaka et al. [15] reported that gastric cancer patients with high levels of NF-κB expression had a lesser overall survival than patients with low levels of NF-κB. The evidence presented above suggested that NF-κB play an important role in the genesis and progression of gastric cancer. Moreover, immunohistochemical staining experiments were conducted to determine in-depth relationship between NF-κB1 expression and SNP site rs4648068. NF-κB1 p50 was found highly expressed in the homozygote GG group compared with the control group. It indicated that the functional site has effects on NFKB1 transcriptional activity and regulates its expression.

The luciferase assay is one of the techniques to study the regulation of gene expression in mammalian cell culture [16,17]. The transcriptional activity could be assessed by calculating the relative luciferase activity of plasmid transfected cells [18]. For luciferase assays, our results showed that the transcriptional activity of NFKB1 was influenced by SNP rs4648068 at gene expression levels. Moreover, the effect on rs4648068 by rs4648065 was also observed at gene transcriptional activity. Our data suggested that rs4648068 GG may upregulate the expression of NFKB1, especially under the influence of rs4648065.

According to our data, the SNP rs4648068 can enhance the transcriptional activity of NFKB1, especially in LPS response. This indicated that the effect of NFKB1 rs4648068 could be influenced by LPS stimulation. Under the LPS stimulation, subsequent reaction occurs through toll-like receptor (TLR)-4-induced signal transduction, which then targets down-stream NF-κB pathway [19]. The activation of NF-κB was affected by LPS stimulation [20]. Similarly, the effect of polymorphism rs4648068 on NF-κB1 expression could be strengthened by LPS stimulation.

Liu et al. reported that various SNPs of cancer related genes are associated with a faster progression of cancers [21]. Polymorphisms in the promoter regions of genes might potentially modulate the gene expression which in turn influence the cell biological activity [18,22]. CHIP assay provided evidence indicating that C/EBPβ can bind to the NFKB1 promoter containing the SNP site [23], We considered that, C/EBPβ bound better to the GG version of NFKB1 promoter and enhanced the transcription of NFKB1, giving rise to the upregulated p50 expression. In addition, NF-κB1 p50 could bind C/EBPβ and reciprocally induce each other’s expression [24], forming a transcriptional positive feedback loop. In fact, C/EBPβ was also confirmed upregulated in the homozygote GG group, accompanied with a higher level of NF-κB1. The mechanism for regulating C/EBPβ expression remains unclear, and will be performed in our future work. C/EBPβ:p50 interaction could induce target genes to influence the cell biological activity [25]. Consequently, the SNP site rs4648068 might influence the cell biological activity by regulating NF-κB1 and C/EBPβ expression.

The NFKB1 polymorphism (rs4648068) was found associated with the cell proliferation and motility in gastric cancer in this study. For the proliferation and invasion assay, the result showed difference in biological activity between transfected cells and control cells. 7901-pGL3-GG cells showed increased ability of growth and invasion, compared with 7901-pGL3-AA cells. For the apoptosis assay, the percentage of apoptosis cells for 7901-pGL3-GG cells decreased significantly, compared with 7901-pGL3-AA cells. The finding suggested that SNP rs4648068 may play a role in regulating gastric cancer cell biological activity.

In this study, we have demonstrated that the NFKB1 polymorphism (rs4648068) is associated with the cell proliferation and motility in gastric cancer. The underlying cellular and molecular mechanisms involve related signaling pathway, such as NF-κB pathway and MAPK pathway. MAPK pathway, as well as NF-κB pathway, is considered the major signaling pathway induced by mediator such as LPS. In the MAPK family, p38 MAPK, c-JNK and ERKs are the most important components. The role of NFKB1 polymorphism (rs4648068) in the regulation of the gene described above should be further studied.

## Conclusions

In summary, the data presented confirmed that the transcriptional activity of NFKB1 was associated with SNP rs4648068. In addition, the functional activity of SNP site was verified by cell biology experiment.
